# Long-term Changes of Temporomandibular Joint Osteoarthritis on Computed Tomography

**DOI:** 10.1038/s41598-020-63493-8

**Published:** 2020-04-21

**Authors:** Hwanhee Song, Jeong Yun Lee, Kyung-Hoe Huh, Ji Woon Park

**Affiliations:** 1 Department of Oral Medicine, Armed Forces Capital Hospital, 177gil-81, Saemeul-ro, Bundang-gu, Seongnam, 13574 Republic of Korea; 2 Temporomandibular Joint Research Center, Seoul Chung-chun Dental Clinic, 15-7, Daehak-ro 10-gil, Jongno-gu, Seoul, 03086 Republic of Korea; 3 Department of Oral and Maxillofacial Radiology, School of Dentistry and Dental Research Institute, Seoul National University, 101, Daehak-ro, Jongno-gu, Seoul, 03080 Republic of Korea; 4 Department of Oral Medicine and Oral Diagnosis, School of Dentistry and Dental Research Institute, Seoul National University, 101, Daehak-ro, Jongno-gu, Seoul, 03080 Republic of Korea

**Keywords:** Outcomes research, Skeleton, Dental diseases

## Abstract

This study aimed to understand long-term changes of the osteoarthritic temporomandibular joint (TMJ) condyle using computed tomography (CT) and to verify its correlation with clinical characteristics of temporomandibular disorders. Eighty-nine patients (152 joints; 76 female, 13 male) who had taken follow-up CTs (mean follow-up period: 644.58 ± 325.71 days) at least once in addition to their initial evaluation were selected. Cross-sectional demographic and clinical data and longitudinal CT images were collected. Data were analyzed by analysis of variance and logistic regression. Overall destructive change index (number of TMJ condyle sections in which destructive change was observed) decreased from 1.56 to 0.66. Improvement was seen in 93 joints (61.2%) and 27 joints (17.8%) worsened. In the pain positive group, both initial and final destructive change index were significantly higher compared to the pain negative group (p = 0.04). Occlusal stabilization splint therapy and nonsteroidal anti-inflammatory drug administration showed a significant effect on improving the prognosis of TMJ osteoarthritis (p = 0.015 and 0.011). In conclusion, TMJ osteoarthritis showed long-term improvement in the majority of cases. TMJ osteoarthritis accompanied by pain showed unfavorable prognosis with additional bone destruction. Occlusal stabilization splint and nonsteroidal anti-inflammatory drug administration were beneficial on the prognosis of TMJ osteoarthritis.

## Introduction

Osteoarthritis (OA) is a debilitating degenerative disorder involving inflammatory conditions and osseous changes of the joints^1,2^. Tissue damage caused by mechanical irritation may lead to the infiltration of inflammatory mediators originating from the synovium into the cartilage, eventually causing defects in cartilage metabolism. Catabolic processes overpower the anabolic capacity of chondrocytes with continued degeneration, tipping the homeostatic balance resulting in progressive cartilage destruction^1,3,4^.

Signs and symptoms of temporomandibular joint (TMJ) OA include pain, movement limitations, clicking and crepitus sounds, and most critically joint deformity that can be identified through radiographic imaging. Osseous changes of TMJ OA manifest as flattening, osteophyte formation, sclerosis, erosion, joint mice, and subchondral bone cysts. Destruction of the TMJ condyle can cause malocclusion and skeletal facial deformity, mainly retrognathism accompanied by anterior open bite and facial asymmetry.

The TMJ condyle is a small bone that is surrounded by various adjacent structures that make visualizing detailed bony status difficult with plain radiography. Computed tomography (CT) images provide an advantageous view of osseous changes by allowing visualization of the bony structure in multiple dimensions with superior reliability and accuracy compared to panoramic radiographs and conventional tomography^5,6^.

The disagreement between the severity of clinical symptoms and radiographic evidence is well described in several previous studies of TMJ OA based on CT results. Pain-related variables were not associated with an increased prevalence of degenerative findings in TMJ tomograms and cone-beam CT (CBCT) findings did not show a consistent correlation with subjective symptoms or bony changes of the condyle^7,8^. On the other hand other studies state that factors such as age and gender have a larger impact on TMJ OA prognosis^7,9^.

Unfortunately only a few studies report on the longitudinal development of TMJ OA based on CT^10–13^. Assessment of clinical and radiographic data focusing on long-term longitudinal bone change could offer a more accurate picture concerning the prognosis of TMJ OA compared to a cross-sectional study. Considering the current lack of a guideline on TMJ OA intervention and the insufficiency of related data long-term longitudinal studies on TMJ OA considering clinical factors are very much called upon.

In this study, the clinical features of TMJ OA patients, longitudinal osseous changes of the TMJ condyle and their interrelationships were analyzed to verify factors and treatment modalities that assist long-term TMJ OA improvement.

## Results

### Demographic and clinical characteristics

The total subject group was consisted of 76 females and 13 males (152 joints) with a mean age of 33.17 ± 17.65 years. TMJ OA was most commonly observed in patients in their 20 s followed by those in their 10 s, 30 s, ≥60 s, 40 s and 50 s in higher order. The mean follow-up period was 644.58 ± 325.71 days. Mean comfortable mouth opening (CMO) and maximum mouth opening (MMO) value was 39.43 ± 10.55 mms and 43.22 ± 9.05 mms respectively. Mouth opening limitation (MOL) (MMO < 38 mm) was observed in 25 subjects (26.97%). Subjective pain was reported by 50 subjects (56.18%). Joint sounds on mouth opening was observed in 26 subjects (29.21%). Centric occlusion (CO)/centric relation (CR) discrepancy was observed in 32 subjects (35.96%). Disc displacement was observed in 52 subjects (34.21%). Stabilization splint therapy was applied to the majority of subjects (72 subjects, 80.90%) and mean treatment duration was 524.49 ± 258.57 days. Intra-articular injection was done in 21 subjects (23.60%). Non-steroidal anti-inflammatory drugs (NSAIDs) were prescribed in 45 subjects (50.56%). Mean duration of NSAIDs prescription was 61.91 ± 42.15 days (Table 1).

**Table 1:** Effect of demographic and clinical factors on TMJ OA prognosis and change of DCI.

Study variables		N (%) or Mean ($${SD})$$	Significance^a^	Change in DCI		
				Initial DCI Mean (*SD*)	Final DCI Mean (*SD*)	Significance^b^
Age (years)		33.17 (*17.65*)	0.733			
Age group	10 s	23 (25.84%)		1.60 (*1.91*)	0.70 (*0.90*)	0.151
	20 s	27 (30.34%)		1.34 (*1.74*)	0.34 (*0.71*)	
	30 s	14 (15.73%)		1.52 (*1.55*)	0.92 (*1.38*)	
	40 s	6 (6.74%)		1.22 (*1.55*)	0.11 (*0.31*)	
	50 s	6 (6.74%)		1.40 (*0.92*)	0.60 (*0.80*)	
	60 s $\le$	13 (14.61%)		2.13 (*1.96*)	1.17 (*1.28*)	
Gender	Male	13 (14.61%)	0.472	1.33 (*1.61*)	0.90 (*1.57*)	0.748
	Female	76 (85.39%)		1.60 (*1.79*)	0.63 (*0.92*)	
F/U period (days)		644.58 (*325.71*)	0.464			
CMO (mm)		39.43 (*10.55*)				
MMO (mm)		43.22 (*9.05*)				
MOL (<38 mm)	No	64 (71.91%)	0.182	1.67 (*1.91*)	0.62 (*1.03*)	0.393
	Yes	25 (26.97%)		1.30 (*1.32*)	0.77 (*1.04*)	
Pain	No	39 (43.82%)	0.119	1.34 (*1.69*)	0.47 (*0.82*)	0.040*
	Yes	50 (56.18%)		1.74 (*1.82*)	0.83 (*1.17*)	
Noise	No	63 (70.79%)	0.156	1.42 (*1.71*)	0.66 (*1.10*)	0.160
	Yes	26 (29.21%)		1.91 (*1.86*)	0.68 (*0.87*)	
CO/CR discrepancy	No	57 (64.04%)	0.032*	1.66 (*1.92*)	0.76 (*1.14*)	0.838
	Yes	32 (35.96%)		1.37 (*1.43*)	0.50 (*0.79*)	
Disc displacement	No	89 (58.6%)	0.763	1.52 (*1.78*)	0.70 (*1.08*)	0.529
	Yes	63 (41.4%)		1.62 (*1.76*)	0.62 (*0.99*)	
Occlusal stabilization splint	No	17 (19.10%)	0.015*	1.76 (*2.05*)	0.76 (*0.90*)	0.490
	Yes	72 (80.90%)		1.51 (*1.69*)	0.64 (*1.07*)	
Intraarticular injection	No	68 (76.40%)	0.057	1.62 (*1.90*)	0.66 (*1.09*)	0.522
	Yes	21 (23.60%)		1.36 (*1.23*)	0.67 (*0.85*)	
NSAIDs	No	44 (49.44%)	0.011*	1.48 (*1.86*)	0.44 (*0.85*)	0.116
	Yes	45 (50.56%)		1.64 (*1.66*)	0.89 (*1.16*)	
Initial DCI			0.000*			

SD, standard deviation; F/U, follow-up ; CMO, comfortable mouth opening; MMO, maximal mouth opening; CO/CR, centric occlusion/centric relation; NSAIDs, non-steroidal anti-inflammatory drugs; MOL, mouth opening limitation; DCI, destructive change index.

^a^Analyzed by logistic regression; ^b^analyzed by repeated measures ANOVA.

^*^p < 0.05.

### Assessment of TMJ OA

The mean value of the initial and final destructive change index (DCI) was 1.56 and 0.66 respectively. Osteoarthritic changes of the TMJ improved in 93 joints (61.2%, 11 male and 82 female joints), worsened in 27 joints (17.8%, 3 male and 24 female joints), and did not change in 32 joints (21.1%, 7 male and 25 female joints). The mean DCI of the improved group decreased from 2.30 to 0.54; male from 1.91 to 0.45 and female from 2.35 to 0.55. The mean DCI of the worsened group increased from 0.33 to 1.37; male from 0.00 to 2.33 and female from 0.38 to 1.25. The difference according to gender was not statistically significant in each TMJ OA prognosis group (Table 2). Initial DCI of the improved group (DCI 2.30) was significantly higher than that of the worsened group (DCI 0.33) (p = 0.004) (Fig. 3).

**Table 2:** TMJ OA prognosis according to change of DCI.

Prognosis	Gender	N (%)	Initial DCI (*SD*)	Final DCI (*SD*)
Worsened	M	3	0.00 (*0.00*)	2.33 (*2.31*)
	F	24	0.38 (*0.58*)	1.25 (*0.67*)
		27 (17.8%)	0.33 (*0.54*)	1.37 (*0.95*)
No change	M	7	1.00 (*2.24*)	1.00 (*2.24*)
	F	25	0.28 (*0.61*)	0.28 (*0.61*)
		32 (21.1%)	0.44 (*1.14*)	0.44 (*1.14*)
Improved	M	11	1.91 (*1.22*)	0.45 (*0.52*)
	F	82	2.35 (*1.84*)	0.55 (*0.98*)
		93 (61.2%)	2.30 (*1.77*)	0.54 (*0.93*)
Total		152 (100%)	1.56 (*1.76*)	0.66 (*1.04*)

DCI, destructive change index; M, male; F, female.

Significance analyzed by repeated measures ANOVA.

Changes between initial and final mean DCI values according to various clinical factors are shown in Table 1 and Fig. 4. Only when the subject group was differentiated based on the presence of subjective pain, the difference in the change of DCI was statistically significant (p = 0.040). Both initial and final DCI were higher in the pain positive group. Initial DCI of pain positive and negative group was 1.74 and 1.34 and final DCI was 0.83 and 0.47, respectively. There were no statistically significant differences in the change of DCI between male and female subjects when distinguished by pain. DCI was changed from 1.91 to 1.45 in the pain positive male group, from 1.74 to 0.75 in the pain positive female group and from 1.08 to 0.42 in the pain negative male group, from 1.36 to 0.47 in the pain negative female group. There were no significant differences according to any other variables including age, gender, presence of MOL, noise, CO/CR discrepancy, presence of disc displacement and treatment modality.

### Factors influencing TMJ OA prognosis

Logistic regression analysis results showed that initial DCI (p = 0.000), presence of discrepancy larger than 2 mms between CO/CR position (p = 0.032), experience of occlusal stabilization splint therapy (p = 0.015), and administration of NSAIDs (p = 0.011) were factors with significant influence on TMJ OA improvement (Table 1). Although the effect of intra-articular injection approached the borderline of significance (p = 0.057), other variables including age, gender, follow-up period, presence of MOL, presence of disc displacement, pain, and noise did not significantly influence the prognosis of TMJ OA.

### Remodeling of condyle with TMJ OA

Total recovery and complete remodeling from the destructive changes of the condyle defined as a previous DCI score ≥1 and then a final DCI score of 0, was seen in 64 joints (42%) in a mean follow-up duration of 646.38 ± 291.23 days. Age, gender, experience of occlusal stabilization splint therapy, administration of NSAIDs, report of subjective pain, and CO/CR discrepancy larger than 2 mms did not show any statistically significant influence on TMJ remodeling in regression analysis.

Although most subjects showed a one directional change of gradual worsening or improvement in TMJ OA, 9 subjects did not. Their DCI once improved and then worsened again.

## Discussion

TMJ OA is a relatively common phenomenon that appears not only in patients complaining of TMD symptoms but it can also be incidentally identified during the course of any dental procedure. Deformity of the TMJ condyle due to OA can be easily confirmed with radiography and diagnosis is becoming more effective with the development of imaging modalities such as computed tomography. However, considering the fact that osseous changes of TMJ OA can eventually lead to irreversible changes in occlusion and facial morphology, information supporting clinical decisions for proper intervention is still insufficient.

OA is generally considered a female predominant^14–16^ and age-related condition. However, interestingly the linear correlation between bone change and age seen in other joints is not observed in the TMJ. TMD is most prevalent in young adults in their 20 s to 40 s and TMJ OA may begin at a very early age^15,17–19^. Previous clinical and histopathological study showed that the mean age of TMJ OA occurrence was 34 years, which is in line with the results of this study^20^. The age and gender distribution of this study did not differ from previous studies on TMJ OA, thus allowing the generalization of our results.

In this study, various cross-sectional demographic and clinical data was analyzed with longitudinal serial CT image sets of TMJ OA. The mean follow-up period was 644.58 ± 325.71 days, which is long enough to allow sufficient assessment of TMJ OA prognosis in a longitudinal manner. Follow-up periods of most other longitudinal studies investigating the osseous prognosis of TMJ OA range from 6 months to 1 year^10–13^. Due to the relatively longer follow-up period, resolution of the destructive changes in the TMJ condyle could be observed in many cases (42% of total evaluated joints). Lei *et al*. reported a 62.7% (42/67) regeneration rate of the TMJ condyle in young adults with early stage OA; 78.1% of the anterior repositioning splint treatment group (25/32) and 48.6% of the control group (17/35)^21^. Such results show that TMJ condyles with erosive surfaces can gradually recover and regain intact cortical lining over a long period of time which generally appears as sclerosis and flattening of the condyle morphology on radiographic imaging. Such processes may be called undestructive remodeling of the TMJ condyle. Without surface erosion or existing subchondral bone cysts, the TMJ condyle can be expected to endure loading that occurs during daily jaw functioning without progressive inflammation^6^.

Various terms have been applied to defining the condition of osseous changes of the TMJ condyle observed on radiographs accompanied by common signs and symptoms of TMD such as pain and dysfunction. According to research diagnostic criteria for TMD (RDC/TMD) guidelines^22^ ostearthrosis and osteoarthritis are both a subtype of degenerative joint disease only distinguished by the presence of pain and dysfunction. However, such differentiation is not well noted in medical literature and both terms have been used interchangeably^6^. Earlier Toller *et al*. also published results on degenerative joint disorder or arthrosis as a type of temporomandibular arthropathy^17^. A number of published studies at that time had used osteoarthrosis synonymously with degenerative joint disease of TMJ. However degenerative joint disorder or osteoarthritis is more commonly used nowadays. In this study we used osteoarthritis in the same vein embracing degenerative joint disease and osteoarthritis. The remodeling group would be in a state more similar to osteoarthrosis defined by RDC/TMD, however the presence of pain was not considered in the differentiation.

The mean period for remodeling to take place in our patients was 646.38 ± 291.23 days. This value is the first to be suggested in literature and could be considered in the clinical evaluation and treatment planning of TMJ OA, but the fact that the true initiation of OA could not be determined should be considered.

Bone changes of the TMJ condyle were qualitatively measured based on visual evaluation of 9 sections of the TMJ condyle. Cevidanes *et al*.^23,24^ suggested a 3 dimensional quantification condylar resorption model technique and Ok *et al*.^11^ used superimposition with 6-imaginary sections to assess the longitudinal bone change in TMJ OA. Although both methods are suitable to assess TMJ OA, the method applied in this study was sufficient to highlight the importance of cortical bone intactness and also more simple allowing less room for error^13,25^. DCI was calculated by counting the sections with destructive changes among the 9 sections of the TMJ condyle and was considered to represent the extent of destructive osseous changes occurring due to TMJ OA. Since we divided the condyle surface into sections and assessed the intactness of each section separately, DCI could accurately reflect the overall extent of destruction better than indirect superimpositioning.

The intactness of the cortical lining may be more important in sustaining the overall health of the TMJ condyle and may reflect the state of the disease more precisely rather than morphological changes occurring as a consequence of bony destruction. Ko *et al*. reported that thickening of the cortex is presumably a response of the subchondral bone to a nonpathological level of pressure^26^. Bony changes in the cortical area may be considered as a radiographic index of the level of pathologic mechanical overloading occurring in the TMJ condyle that may be reduced through treatments such as occlusal splints. A condyle with sound cortical surfaces can bear overloading better and not undergo a chronic inflammatory process^6^.

Lee *et al*. first introduced the 9-imaginery section method and its reliability was proven in a previous study^13,25^. In this study the landmarks of Lee’s method were modified to enhance practicality by using tangent lines to the most prominent point in the contour of the condyle instead of the squamotympanic fissure and the apex of the eminence. The squamotympanic fissure can easily be skipped on tomographic images according to slice thickness and the location of the apex of the eminence can be affected by erosive changes occurring with OA. A preliminary study was conducted to verify the reliability of this modified method. CT images were evaluated for TMJ OA based on the modified method three times with a two-week interval by a single examiner who is a trained TMD and orofacial pain specialist with more than 7 years of clinical experience. The reader was blind to all other clinical data. The Cohen’s kappa value was 0.739 (p < 0.005 in all comparison among the 3 readings) showing substantial reliability.

The TMJ condyle showed recovery in the majority of subjects and the mean extent of destructive change decreased during the total follow-up period. TMJ OA is known as a self-limiting disease that shows spontaneous recovery. This process is mediated through the body’s immune system and the duration of progress can be shortened through optimal intervention^27–29^. The results of this study showed that the initial extent of bone change was more severe in the improved group (initial DCI 2.30) compared to the worsened group (initial DCI 0.33). This implies that severe destruction of the TMJ condyle observed on initial radiographic imaging does not necessarily reflect more aggressive disease activity and even relatively severe destructions may show improvement while initially mild osteoarthritic changes can progressively worsen. Logistic regression analysis also showed that high initial DCI value is a significant index for prediction of better TMJ OA prognosis. Untreated TMJ OA may have a natural course of disease with a predetermined time of progression and the subjects of the worsened group may have been examined in the early stage of disease while the joints in the improved group were examined around the peak of the disease progression^13^.

The fact that a TMJ condyle which initially shows normal to mild OA change can later show more bony destruction warrants periodic follow-up CT imaging even with a patient receiving regular treatment. According to the results of this study a 2 year follow-up period for TMJ OA may be suggested and considered in treatment planning as the mean disease span for progression and termination was 646.38 ± 291.23 days to observe no further change in OA severity. Considering OA as a structural re-adaptation process to enable normal function with certain discrepancies between functional loading and the natural shape of the TMJ condyle, structural change may recommence unless contributing factors causing excessive loading are eliminated. So behavioral therapy to control unfavorable contributing factors must be persistently applied to ensure the successful management of TMJ OA.

Most demographic and clinical factors, such as age, gender, range of mouth opening, presence of joint noise and accompanying disc displacement showed no significant relationship with osseous changes in TMJ OA through repeated measures ANOVA which is in line with other previous studies^7,8,13,30^. While other studies state that factors such as age and gender are closely related to TMJ OA prognosis^7,9^.

The results of this study showed that disc displacement did not have a significant effect on TMJ OA prognosis and there was no significant difference in the change of DCI. There have been a number of arguments related to the relationship between disc displacement and TMJ OA reflecting the close relationship between the two conditions^28,31,32^. However, the data until now does not directly support causality and the sequential or causal relationship of disc displacement with the TMJ OA is yet to be established. The discrepancy of the results of this study from others may have resulted from the difference in diagnostic approaches or definitions of disc displacement. Disc displacement was judged clinically following RDC/TMD guidelines^22^ of proven reliability in a longitudinal manner rather than cross-sectionally and this should be considered in comparing results with comparable studies.

Only the presence of subjective pain at the initial examination showed a significant relationship with the difference in change of DCI between groups in this study. In the pain positive group, both initial and final DCI were significantly higher compared to the pain negative group. Patients with TMJ OA accompanied by pain may show additional osseous destruction so the pain level must always be evaluated at the initial diagnostic process and those with subjective pain should be treated more aggressively from the beginning of treatment to gain favorable results. The mechanism in which subjective pain is related to destructive bone change is yet to be elucidated, however active interventions to control initial pain should be considered. Inflammatory cytokines that are known to directly evoke pain may also contribute to the additional osseous changes of the TMJ condyle sharing a common pathway between pain transmission and bone resorption^33–35^. Cevidanes *et al*. also revealed that the extent of resorptive changes in the TMJ OA condyle were closely related to pain severity and duration^23^.

Meanwhile, it is interesting that CO/CR discrepancy showed to be another factor that significantly influences TMJ OA prognosis. The subjects with CO/CR discrepancy showed a favorable prognosis with their osseous changes accompanied by more active remodeling. The relationship between CO/CR discrepancy and TMD has been a controversial issue until now. Some suggested CO/CR discrepancy could lead to TMJ arthralgia, myalgia, disc displacement, and even TMJ OA^36–39^. And others have contended that an association between CO/CR discrepancy and TMD could not be established^40–44^. Though CO/CR discrepancy appears to be a statistically significant factor that influences the prognosis of TMJ OA, this does not directly support a cause-and-effect relationship between CO/CR discrepancy and TMJ OA. The initial DCI of the improved group was significantly higher so occlusal disharmony or CO/CR discrepancy may have been a mere consequence of this state^36^.

Several treatment modalities including behavioral modification, physical therapy, medication, occlusal splint therapy, intra-articular injection, and surgical procedures have been applied for the management of TMJ OA. Their treatment efficacy in improving signs and symptoms of TMD have been sought through previous studies^11,27,45–52^. However, studies looking into the treatment efficacy focusing on bone changes of TMJ OA are scarce. The logistic regression analysis results of this study revealed that occlusal stabilization splint therapy and NSAIDs had a significant influence on TMJ OA prognosis. Both modalities are currently the treatment of choice for TMJ OA and the results support their continued application.

Though intra-articular injection did not show a statistically significant effect on TMJ OA prognosis the significance level was relatively high (p = 0.057). Intra-articular injections are known to be effective in controlling pain of TMJ OA^52^. Lavage with intra-articular injections are beneficial by removing inflammatory cytokines associated with not only pain but also osseous changes, thus resulting in favorable TMJ OA prognosis^53,54^. However, the timing of the injection itself may be a crucial factor in determining the effects of the treatment and further well controlled prospective studies are necessary to establish a guideline. The fact that intra-articular injection was applied to those complaining of refractory pain and not responding to conservative treatment including occlusal stabilization splint and medication in this study may have influenced the results. For further investigations, injection count and type of drugs injected should be controlled though the efficacy between hyaluronic acid and corticosteroid on TMJ OA showed no significant differences^55^. The patients who had intra-articular injections were also less in number than those with occlusal stabilization splint or administration of NSAIDs, meaning non-homogeneity with other OA treatment groups.

Reducing general inflammation level by NSAIDs could relieve symptoms of TMJ OA and may consequently improve osseous changes by reducing prostaglandin level, an important mediator of inflammation^34,35,51^. Diclofenac sodium which was the only NSAIDs prescribed in this study has been known for its effectiveness in the management of TMD of arthrogenous origin^56^. Further studies to establish a guideline including dosage and administration regimen of NSAIDs in TMJ OA are warranted. Occlusal splints are known to relieve mechanical overloading and guard the condyle from recurrent hypoxia^57^. Favorable treatment responses have been verified in TMJ OA^10,21,51,58–60^. By combining the 2 treatment modalities it could be possible to physically reduce excessive loading on the condyle and chemically remove inflammatory mediators leading to efficient improvement of bony changes occurring in the TMJ condyle.

Osteoarthritic changes of the TMJ condyle were evaluated in a longitudinal manner showing that destructive changes of the TMJ condyle due to TMJ OA improved in the majority of cases. Restoration of bony structure by regaining cortical intactness in TMJ OA could be observed in approximately 2 years on average and occlusal splint therapy and NSAIDs appeared to be beneficial. Initially mild destructive bone changes may progress more destructively so periodic follow-up imaging is essential. TMJ OA accompanied by pain showed unfavorable osseous prognosis. Such findings should be considered in the diagnosis and personalized treatment planning of TMJ OA.

Understanding the long-term osseous change in TMJ OA and assessing factors that may influence this process leads to information that may assist the selection of appropriate intervention methods and treatment timing that will eventually result in better prognosis of TMJ OA.

## Methods

### Subjects

Consecutive patients who visited the Orofacial Pain Clinic of our dental hospital complaining of TMD symptoms and showed bone change of the TMJ condyle on CT from January, 2010 to January, 2015 were reviewed. Among the initial study group, 89 patients (152 joints excluding 26 joints which showed no bone change on any of the CT image sets) who had taken follow-up CTs at least once or more were selected for final analysis. There were 76 women and 13 men in the final study group and the mean age was 33.17 ± 17.65 years. The mean CT assessment interval was 376.61 ± 94.49 days. Subjects with a history of orthodontic treatment, orthognathic surgery, macrotrauma, fracture, and systemic diseases that could cause joint deformity such as rheumatoid arthritis were excluded. This study was approved by the Institutional Research Board of Seoul National University Dental Hospital. The IRB authorized exemption of informed consent from the subjects (#ERI18009). All procedures performed involving human participants were in accordance with the ethical standards of the institutional research committee and with the 1964 Helsinki declaration and its later amendments or comparable ethical standards.

### Clinical evaluation

Clinical assessment was done according to the RDC/TMD^22^. Clinical indices including range of mouth opening, subjective pain level, presence of joint noises, discrepancy between CO and CR position and presence of disc displacement were assessed by 2 oral medicine specialists. Presence of disc displacement was diagnosed according to RDC/TMD guidelines^22^. Disc displacement was categorized into 2 levels; 0, no disc displacement and 1, disc displacement present.

Range of mouth opening was measured as the shortest distance between the upper and lower incisors at the midline in millimeters. MMO was measured as the amount of spontaneous maximum mouth opening range regardless of the presence of pain and CMO was measured as the maximum amount of spontaneous mouth opening range without pain. Those with a MMO less than 38 mms were included in the MOL group. Subjects who reported TMJ pain at rest and/or during function were included in the subjective pain positive group. The presence of joint sounds including clicking and crepitus on jaw movement were also recorded. The distance of sliding between CO and CR position when guided by a TMJ specialist was also measured both antero-posteriorly and laterally in millimeters. A distance more than 2 mms between CO and CR in any direction was regarded as showing a positive CO/CR discrepancy.

Treatment modalities applied to the patients included occlusal stabilization splint therapy, intra-articular injections using hyaluronic acid and triamcinolone, and medication including NSAIDs. The occlusal stabilization splint was made of hard acrylic resin with even bilateral occlusal contact with the premolars and molars in CR position with a 2 mm thickness in the molar area. The mean duration of occlusal stabilization splint therapy was 524.49 ± 258.57 days. Intra-articular injection was done with single needle technique, which is relatively simple compared to the conventional 2-needle technique and reportedly more comfortable for the patients^61^. A single needle was inserted into the upper joint space followed by lavage and administration of hyaluronic acid (Shin Pung Pharm. CO., Ltd, Korea) and triamcinolone (Shin Pung Pharm. CO., Ltd, Korea). The type of prescribed NSAIDs included diclofenac sodium only by oral administration. The mean duration of medication prescription was 61.91 ± 42.15 days.

### Computed tomography assessment

#### Acquisition of CT images

CT images were acquired using SOMATOM Sensation 10 (Siemens, Erlangen, Germany) with 0.75 mm slice collimation. Patients were positioned in a supine position during image acquirement and corrected sagittal, coronal, and axial images of the TMJs were reconstructed along the true axes of the mandibular condyle at a slice thickness of 1–2 mm.

#### Cross-sectional assessment of TMJ OA

TMJ OA diagnosis was based on identification of typical osteoarthritic changes of the TMJ articular surface on CT images. One oral medicine specialist evaluated the presence of the following imaging characteristics known to be related to TMJ OA: (1) flattening, loss of the rounded contour of the articular surface; (2) erosion, loss of continuity of the articular cortical bone; (3) osteophytes, marginal bony outgrowths of the condyle; (4) sclerosis, increased thickness of the cortical plate in the load-bearing areas compared to the non-load bearing adjacent areas; and (5) subchondral cyst, cavity formation below the articular surface that deviates from normal bone marrow pattern^62,63^.

**Figure 1:**
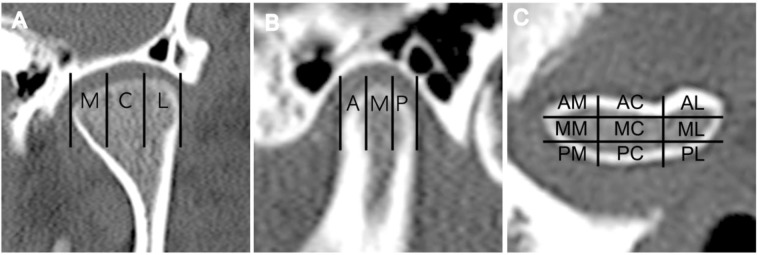
Evaluation approach for osteoarthritic changes of the mandibular condyle. (**a**) The condylar surface was divided into three sections in the medio-lateral direction between the tangent line on the most medial and lateral point. (M, medial; C, central; L, lateral). (**b**) The condylar surface was divided into three sections in the antero-posterior direction between the tangent line on the most anterior and posterior point. (A, anterior; M, middle; P, posterior). (**c**) The condylar surface was divided into nine imaginary sections. (AM, antero-medial; AC, antero-central; AL, antero-lateral; MM, mid-medial; MC, mid-central; ML, mid-lateral; PM, postero-medial; PC, postero-central; PL, postero-lateral).

Joints with condyle surfaces showing erosion and subchondral cyst formation on CT images were considered as undergoing destructive changes. Flattening, sclerosis and osteophyte formation not accompanied by erosion or subchondral cysts were regarded as undestructive types of TMJ OA. Consensus is yet to be reached whether osseous changes with low destructivity such as flattening, sclerosis, and osteophyte formation with stable integrity of cortical lining should be diagnosed as OA or not^6,64^. To determine the extent of OA destruction the condyle surface was divided into three sections in the anteroposterior direction between the tangent line of the most anterior and posterior point of the condyle. The condyle was divided into three sections again in the mediolateral direction between the tangent lines of the most medial and lateral point of the condyle on corrected coronal views, resulting in nine sections of the entire condyle surface following the modified method of Lee *et al*. (Fig. 1)^13^. Destructive change index (DCI) was calculated as the number of sections in which erosion or subchondral cyst formation could be observed to represent the extent of destructive change by TMJ OA.

#### Longitudinal assessment of TMJ OA

**Figure 2:**
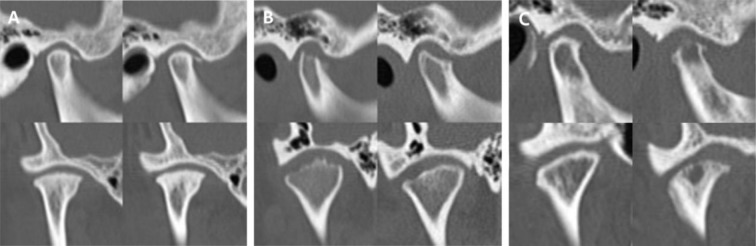
The three groups based on longitudinal TMJ OA bone change. (**a)** No change group; (**b**) Improved group; (**c**) Worsened group. The left side shows the initial and the right side shows the final CT examination in each image set.

Longitudinal bone change of TMJ OA was evaluated as the change of DCI between the first and follow-up CT image set. The prognosis of TMJ OA was classified into the three following groups: no change, no change of DCI; improved, a decrease in DCI; worsened, an increase in DCI (Fig. 2). Cases showing total recovery that is DCI value of 0 on the final CT images from DCI value of 1 or more on the previous CT images were categorized as the remodeling group^13^. The single CT examiner was blind to any other clinical information concerning the subject. In subjects with 3 or more serial follow up CTs, prognosis grouping was done according to the change between the initial and final DCI.

**Figure 3:**
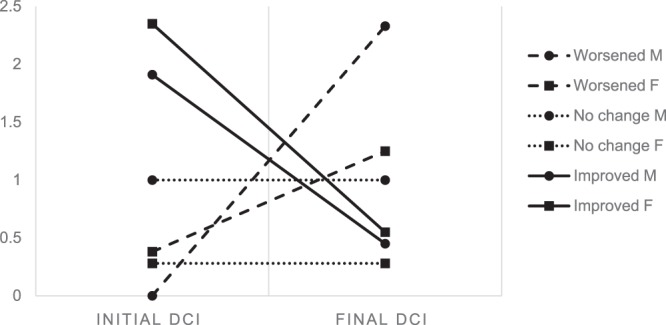
Change of DCI according to prognosis of TMJ OA. DCI, destructive change index. *P* = 0.004, Significance analyzed by repeated measures ANOVA. SPSS 21.0 software (SPSS Inc., Chicago, IL, USA) https://www-01.ibm.com/support/docview.wss?uid=swg24032236.

**Figure 4:**
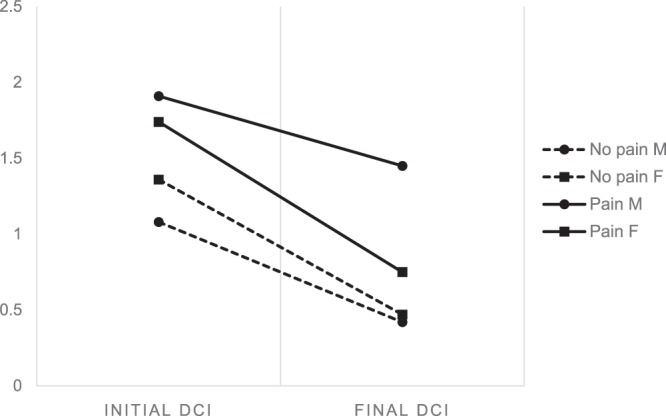
Change of DCI according to presence of subjective pain. DCI, Destructive change index. *P* = 0.04, Significance analyzed by repeated measures ANOVA. SPSS 21.0 software (SPSS Inc., Chicago, IL, USA) https://www-01.ibm.com/support/docview.wss?uid=swg24032236.

### Statistical analysis

Frequency of demographic and clinical data was analyzed by Chi-square test. Changes of DCI according to clinical variables were analyzed by repeated-measures ANOVA. To assess significant factors influencing the prognosis of TMJ OA logistic regression analysis was used. All statistical analysis was performed using SPSS 21.0 software (SPSS Inc., Chicago, IL, USA). Results were considered statistically significant at a level of p < 0.05.
